# eGFR in CSF-flow disorders - a representation of comorbid state or an element of the underlying pathophysiology?

**DOI:** 10.1186/2045-8118-12-S1-P17

**Published:** 2015-09-18

**Authors:** Patricia Haylock-Vize, Claudia Craven, Edward Dyson, Chari Aswin, Samir A Matloob, Andrew Stevens, Simon D Thompson, Syed N Shah, Tarek Moustafa, Neekhil A Patel, Huan Wee Chan, Jinendra Ekanayake, Ahmed K Toma, Laurence D Watkins

**Affiliations:** 1 The National Hospital for Neurology and Neurosurgery, UK

## Introduction

All cause mortality increases with lower levels of estimated glomerular filtration rates. As part of a comorbidity focus, we assessed the eGFR in CSF-flow disorder patients guided by NICE recommendations, current literature recognising a relative hazard ratio per 15mls/min decrease in eGFR of 1.04[1] and consideration of the 2010 Chronic Kidney Disease Epidemiology Collaboration eGFR categories.

## Methods

182 CSf-flow disorder patients who had eGFR measurement were grouped into the respective subtypes; IIH (n=41), NPH (n=72), CSF hypovolaemia (n=7) and other causes (n=45). Others included those investigated for NPH but found not to be suitable for shunt.

Estimated GFR was categorised as per NICE guidelines; (ml/min/1.73m) in line with current clinical laboratory facilities;

>90 normal high, 

60-89 mild reduction related to normal range for a young adult, 

45-59 mild-moderate reduction, 

30-44 moderate-severe reduction, 

15-29 severe-reduction, 

<15 kidney failure.

The eGFR's were recorded and the mean calculated for each subgroup.

## Results

IIH cohort; mean eGFR 77.73;

26 patients >90 normal high, 14 patients in 60-80 mild reduction, and 1 patient 45-59 mild-moderate category.

Primary NPH; mean eGFR 73.32mls/min; 18 patients >90 normal high, 35 patients 60-89 mild reduction, 17 patients 45-59 mild-moderate and 2 patients 30-44 moderate-severe category.

Secondary NPH; mean eGFR 81.94mls/min; 16 patients 60-89 mild reduction range and 1 patient 45-59 mild-moderate category.

Orthostatic HA; mean eGFR 87.25mls/min; 5 patients >90 normal high and 2 patients 60-89 mild reduction.

Other cohort; mean eGFR 76.96mls/min; 22 patients >90, 21 patients 60-89 mild reduction and 2 patients 45-59 in the moderate-severe reduction.

Of the 182 patients, 71 patients had high normal eGFRs >90mls/min representing 39% of patients. 48% fell into the 60-89 mild reduction range, 11.5% into the 45-59 mild-moderate reduction range and 1% into the moderate-severe reduction range.

## Conclusions

A reduced estimated GFR is commonly seen in the CSF-flow disorders patient cohorts. Given the emphasis towards comorbidity factors influencing surgical outcomes, this acts as a marker of comorbidity and physiological reserve for surgical tolerance and post-operative performance and should therefore be taken into consideration.

The altered eGFR could represent the comorbid state of the patient or it could form an element of the underlying pathophysiology of CSF-flow disorders or indeed both. Larger studies are required with more translational medical research models.
